# Culture Positive Cases of Ophthalmia Neonatorum in a Tertiary Care Centre of Nepal: A Descriptive Cross-sectional Study

**DOI:** 10.31729/jnma.5044

**Published:** 2021-04-30

**Authors:** Sabina Shrestha, Sunil Raja Manandhar, Om Krishna Malla

**Affiliations:** 1 Department of Ophthalmology, Kathmandu Medical College and Teaching Hospital, Sinamangal, Kathmandu, Nepal; 2 Department of Pediatric, Kathmandu Medical College and Teaching Hospital, Sinamangal, Kathmandu, Nepal

**Keywords:** *antibiotic sensitivity*, *causative organisms*, *ophthalmia neonatorum*

## Abstract

**Introduction:**

Ophthalmia neonatorum although runs a benign course mostly, sometimes may progress to sight threatening complications. The study was conducted to find the prevalence of culture positive cases of opthalmia neonatorum.

**Methods:**

It was a descriptive cross-sectional study conducted at a tertiary care center from January to December 2019. Ethical clearance was obtained from institutional review committee of Kathmandu Medical College. Convenience sampling was done. All data were entered into excel and, then for analysis, exported to Statistal Package for Social Sciences version 21. Point estimate at 95% Confidence Interval was calculated along with frequency and proportion for binary data.

**Results:**

The prevalence of culture positive cases of opthalmia neonaturum is 10 (55.55%) (32.61-78.49 at 95% Confidence Interval). The causative organisms were coagulase negative *Staphylococcus* 4 (40%), *Staphylococcus aureus* 3 (30%), *Klebsiella* 2 (20%) and *Pseudomonas* 1 (10%). Culture sensitivity of the isolated organisms were different according to the patient even in case of the same organism. Vancomycin 7 (70%) was the most sensitive antibiotic followed by Ciprofloxacin 6 (60%), Amikacin 5 (50%) and Cloxacillin 5 (50%) while Azithromycin 1 (10%), Cefixime 1 (10%) and Cotrimoxazole 1 (10%) were the least sensitive.

**Conclusions:**

*Staphylococcus* species was the most common organism isolated from neonates with ophthalmia neonatorum and vancomycin was the most sensitive antibiotic.

## INTRODUCTION

Conjunctivitis is inflammation of the conjunctiva presenting with conjunctival congestion, discharge and chemosis.^1^ Ophthalmia neonatorum is an acute infection of the conjunctiva occurring within the first four weeks of life affecting 1.6 to 12% of all newborns.^2-4^ It can be caused by chemical, bacterial or viral processes.^3^ It was the primary cause of neonatal blindness before 1880s and the term ophthalmia neonatorum was used only for the conjunctivitis caused by Neisseria gonorrhoeae.^5,6^

Ocular prophylaxis with 2% silver nitrate resulted in decrease in incidence of gonococcal conjunctivitis among neonates from10% to 0.3%.^5,7^ However, silver nitrate can cause chemical conjunctivitis and the fall in practice of ocular prophylaxis in some countries in developed world has resulted in the reemergence of sight-threatening infections.^8^ Ocular prophylaxis should remain the standard of care;^5^ however, debate and controversy have emerged around this issue.^9^

The study was conducted to determine the prevalence of culture positive cases of Ophthalmia neonatorum.

## METHODS

It was a descriptive cross-sectional study conducted in Kathmandu Medical College teaching hospital over a period of one year from January 2019 to December 2020. Ethical clearance was obtained from Institutional Review Committee of Kathmandu Medical College (Ref:150320198) and informed consent was taken from the parents.

Infants up to 28 days of life presenting to the outpatient department of ophthalmology with watering or discharge from the eyes with no other causes for the same, with the parents consenting for the study were enrolled. History was taken from the parents and clinical examination of the child was done and conjunctival swab was sent for culture and sensitivity test under all aseptic precautions. Especially designed proforma was used for recording the patient demographics, history, comprehensive clinical examination findings and investigation results. Since ophthalmia neonatorum patients were very less in Kathmandu Medical College Teaching Hospital in the year 2018 also, we have included all the neonates meeting the inclusion criteria during the study period. Convenience sampling was done and sample size was calculated using the formula,

$$\begin{array}{ll} \text{n} & \text{= }{\text{Z}}^{\text{2}}\times \text{p}\times \text{(1}-\text{p)}/{\text{e}}^{\text{2}}\\ & \text{= }1.96^{\text{2}}\times 0.5\times 0.5/\text{0}{\text{.05}}^{\text{2}}\\ & \text{= }385 \end{array}$$

Adjusted sample size for finite population,

$$\begin{array}{ll} {\text{n}}_{\text{o}} & \text{= (nN)}/[\text{N+(n}-\text{1)}]\\ & \text{= }385/\{1+(385-1)/\text{18\}}\\ & \text{= }18 \end{array}$$

where,

Z = 1.96 at 95% Confidence Interval.
p = prevalence of femoral head necrosis 50% for maximum sample size.
e = margin of error 5 %.
n_o_ = adjusted sample size for finite population
N= Finite Population i.e 18

All data were entered into excel and, then for analysis, exported to Statistical Package for Social Sciences version 21. The collected data were checked for completeness and then processed. Point estimate at 95% CI was calculated and data were expressed in percentage and frequency as and when needed.

## RESULTS

Out of total 18 opthalmia neonatrum cases, 10 (55.55%) (32.61-78.49 at 95% Confidence Interval) were culture positive. Among the participants, male to female ratio was 1:1. Fifty percent of the babies were delivered normally while 50% underwent caesarean section in Kathmandu medical college. Majority of the neonates (94%) had bilateral involvement.

**Figure 1.**
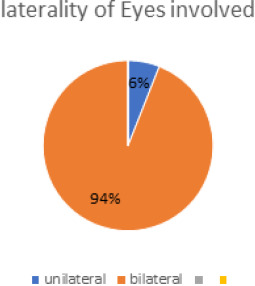
Distribution of patients according to laterality of eye involved.

Ten (55.55%) of the neonates presented within 48 hours (Table 1) and 10 (55.55%) had onset of conjunctivitis within 48 hours of life (Table 2).

**Table 1:** Distribution of time of presentation.

Time of presentation	n (%)
Within 48 hours	10 (55.6)
4-5 days	5 (27.7)
After 10 days of life	3 (16.7)
Total	18 (100)

**Table 2:** Distribution of onset of conjunctivitis.

Onset of conjunctivitis	n (%)
From birth -48 hours	10 (55.55)
4-5 days	4 (22.22)
7-10 days	2 (11.11)
More than 10 days	2 (11.11)
Total	18 (100)

**Figure 2.**
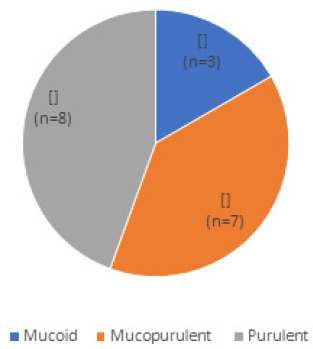
Distribution of type of discharge.

Purulent discharge was the most common type of discharge (Figure 2). Pseudo-membrane was present in 5 cases (28%). However, cornea was not involved in any of the cases. Culture of conjunctival swab was positive in 10 (56%) of conjunctival swab. The causative organisms were coagulase negative staphylococcus (40%), staphylococcus aureus (30%), klebsiella (20%) and pseudomonas (10%) (Table 3).

**Table 3:** Distribution of causative organism among culture positive cases.

Causative Organism	n (%)
Coagulase Negative Staphylococcus	4 (40)
Staphylococcus Aureus	3 (30)
Klebsiella	2 (20)
Pseudomonas Aeruginosa	1 (10)
Total	10 (100)

Culture sensitivity of the isolated organisms were different according to the patient even in case of the same organism.

**Table 4:** Overall antibiotic sensitivity of the microorganisms isolated.

Antibiotics	Sensitive n (%)	Resistant n (%)
Ciprofloxacin	6 (60)	4 (40)
Imipenem	2 (20)	8 (80)
Piperacillin	3 (30)	7 (70)
Vancomycin	7 (70)	3 (30)
Amikacin	5 (50)	5 (50)
Ceftriaxone	3 (30)	7 (70)
Cloxacillin	5 (50)	5 (50)
Cefixime	1 (10)	9 (90)
Amoxiclav	4 (40)	6 (60)
Azithromycin	1 (10)	9 (90)
Levofloxacin	3 (30)	7 (70)
Gentamicin	3 (30)	7 (70)
Co-trimoxazole	1 (10)	9 (90)
Cefotaxime	3 (30)	7 (70)

Out of ten culture positive organisms, vancomycin was the most sensitive antibiotic accounting for 70% followed by ciprofloxacin (60%), amikacin (50%) and cloxacillin (50%). Azithromycin (10%), cefixime (10%) and clotrimoxazole (10%) were the least sensitive.

## DISCUSSION

Infectious conjunctivitis occurs in 12% of neonates and in 23% of neonates in developing countries.^10,11^ The characteristics of high risk group for ophthalmia neonatorum according to World Health Organization include: pregnant women who have had recent sexual contact with a partner with any sexually transmitted disease, pregnant women with vaginal discharge or dysuria, pregnant women with sexually transmitted disease and women who are pregnant for the first time and have multiple partners.^12^

In the present study, males and females were equally affected. However, most of the studies have shown male preponderance^13-16^ The male gender as a risk factor for increased neonatal infection has been attributed to Y-gene.^15^

The mean age of onset of conjunctivitis was 6.1 days of birth in a study by Khosdel A, et al. before 7 days of birth in a study by Mohile M, et al and 3.7 days in a study by Soltanzadeh M H, et al.^13,14^ In the present study, the mean age of onset of conjunctivitis is 3.6 days of life. However, the mean age of presentation was 13.4 days of life.

Bilateral involvement was seen in most of the cases (94%) in the present study which is similar to the study done by Abolfazl Afjeiee S, et al.^16^ but contradictory to other studies.^13,14^

In a study by Khosdel A, et al., also staphylococcus species was the most common organism isolated which accounted for 44.4%,^17^ Usually, the infectious organism contaminates the neonates through direct contact during passage through the birth canal. However, the infection is also known to reach the uterus so that child delivered by caesarean section can also be affected, more so in cases of prolonged rupture of membrane at the time of delivery.^18^ In the present study also 60% of the mothers with culture positive babies for ophthalmia neonatorum had undergone caesarean section while 40% had delivered normally. However, among the total participants, delivery of the babies was by normal vaginal route and caesarean section is equal in proportion. It should also be taken into consideration that sometimes the newborns can infection from their immediate surroundings which could be due to unhygienic handling of the newborn by attendants after birth.

According to study by Osler HB and Forster RK, pseudomembranes or true membranes may occur and lead to scarring if left untreated.^19,20^ In our study also, 28% of the neonates had pseudomembrane. However, cornea was not involved in any of the cases.

Due to difference in socioeconomic conditions, standard of maternal health care, prophylactic program, hygienic conditions during labor and postnatal exposure to microorganisms, the pathogens accounting for neonatal conjunctivitis vary.^15^

In a Nairobi hospital with no ocular prophylaxis for ophthalmia neonatorum, the incidence was found to be 23.2 per 100 live births among which gonococcal and chlamydial ophthalmia neonatorum incidence was 3.6 and 8.1 per 100 live births respectively.^10^ In vertically transmitted ophthalmia neonatorum, staphylococcus aureus was the most common organism in Argentina, Hong Kong and United Arab Emirates while chlamydia trachomatis was the most common pathogen in China, Germany, Kenya, Thailand and united States.^21-27^

Conjunctival cultures are negative in up to 25% of babies with neonatal conjunctivitis according to Scott R Lambert.^28^ However in our study, it was negative in 44% cases.

In most studies, the most common pathogen isolated in cultures from neonates is staphylococcus aureus.^29,30^ Even other gram-positive organisms like Staphylococcus epidermidis, streptococcus viridens and Streptococcus pneumoniae were isolated. Similarly, gram negative organisms like enterococcus, Escherichia coli, Serratia species and Pseudomonas species are responsible for a lesser percentage of cases.^31^

In a study by Khosdel A, et al. also staphylococcus species was the most common organism isolated which accounted for 44.4% followed by chlamydia trachomatis (13.6%), streptococcus pneumoniae (13%), E. coli (9.9%), enterobacter (7.4%), Klebsiella (3%), H. influenza (1.8%) and pseudomonas (1.2%).^17^ Similarly, in studies conducted by Lyamu E and Ebeigbe J A, staphylococcus aureus predominated as the cause of ophthalmia neonatorum.^32,33^ Staphylococcus accounted for the most common causative organism also in a study by Vincent NJ, et al. (30.2%) and by Soltanzadeh MH, et al. (34.5%).^34,14^ In a case control study, out of 200 cases and 200 control, the most common pathogen isolated was Staphylococcus aureus in both cases (69.7%) and in controls (48%). This can be due to the fact that staph aureus is one of the common commensals of the eye.^35^

In the present study also, the most common organism isolated is staphylococcus, followed by klebsiella and pseudomonas. Streptococcus is isolated in none of the cases in the present study which could be due to the small sample size. And no cases of neisseriagonorrhoea were isolated. In studies done by Mohile M et al, ShireenGul Set et al. and Perera J, Withana N et al also N. gonorrhea was not isolated.^13,15,36^ One of the factors relevant for the absence of N. gonorrhea may be due to the fact that the study was conducted in urban area of Kathmandu where the mothers are screened for sexually transmitted diseases during antenatal period.

The limitation of the study is small sample size and the study is conducted in the hospital setting only.

## CONCLUSIONS

Culture of conjunctival swab was positive in more than half of cases of ophthalmia neonatorum in which staphylococcus species was the most common organism isolated from neonates. Vancomycin was the most sensitive antibiotic sensitive against majority of the organisms detected. Culture sensitivity showed variable sensitivity of the drugs even in case of the same organism isolated. Hence, we would like to recommend neonates presenting with signs of conjunctivitis should have the conjunctival swab sent for culture and sensitivity and treated accordingly.
